# Reporting of paediatric osteoporotic vertebral fractures in Duchenne muscular dystrophy and potential impact on clinical management: the need for standardised and structured reporting

**DOI:** 10.1007/s00247-023-05805-4

**Published:** 2023-12-11

**Authors:** H. Martin, A. Henderson, R. Allen, A. M. Childs, J. Dunne, I. Horrocks, S. Joseph, J. K. Kraft, K. Ward, T. Mushtaq, A. Mason, A. Kyriakou, S. C. Wong

**Affiliations:** 1 Department of Paediatric Endocrinology, Royal Hospital for Children, 1345 Govan Road, Glasgow, G51 4TF UK; 2 Department of Paediatric Neurology, Leeds Teaching Hospital NHS Trust, Leeds, UK; 3 Department of Paediatric Radiology, Royal Hospital for Children, Glasgow, UK; 4 Department of Paediatric Neurology, Royal Hospital for Children, Glasgow, UK; 5 Department of Paediatric Radiology, Leeds Teaching Hospital NHS Trust, Leeds, UK; 6 Department of Paediatric Endocrinology, Leeds Teaching Hospital NHS Trust, Leeds, UK; 7 Department of Paediatric Endocrinology, Makarios Children’s Hospital, Nicosia, Cyprus; 8 School of Medicine, Dentistry and Nursing, University of Glasgow, Glasgow, UK

**Keywords:** Children, Diagnostic imaging, Dual-energy X-ray absorptiometry, Duchenne muscular dystrophy, Radiography, Vertebral fracture

## Abstract

**Background:**

In boys with Duchenne muscular dystrophy (DMD), initiation of bisphosphonate is recommended upon identification of moderate or severe vertebral fractures, even if asymptomatic. Clear radiological reporting is important for consistency of clinical interpretation and management.

**Objectives:**

To audit radiology reports of spine imaging for vertebral fracture assessment in DMD, and assess potential impact on diagnosis and management.

**Materials and Methods:**

Lateral thoracolumbar spine imaging (71 lateral spine radiographs and 13 lateral dual energy absorptiometry spine image) in 84 boys with DMD performed across two centres. Anonymised radiology reports by paediatric radiologists were circulated to two neuromuscular clinicians and two endocrinologists. Clinicians determined if there was vertebral fracture, no vertebral fracture, or unclear interpretation. Endocrinologists also determined if bisphosphonate was indicated. A single observer (a clinician with expertise in vertebral fracture assessment) performed vertebral fracture assessment in 37 images and re-reported using a structured format. Structured reports were re-circulated to the four clinicians to re-evaluate the degree of concordance in clinical diagnosis of vertebral fracture and treatment decisions with bisphosphonate.

**Results:**

The term “fracture” was used in 25/84 (30%) radiology reports and only in 8/43 (19%) with description of vertebral body abnormalities. Fracture grading was included in 7/43 (16%) radiology reports. Diagnostic concordance by the clinicians was noted in 36/84 (43%). Unclear interpretation was noted in 22% to 51% based on radiology reports. No unclear interpretation was noted with structured reports. Complete diagnostic (37/37, 100%) and treatment (37/37, 100%) concordance was noted with the structured reports, whereas complete diagnostic and treatment concordance was noted in only 16/37 (43%) and 17/37 (46%) of the radiology reports, respectively.

**Conclusion:**

Only a third of radiology reports of spine imaging in DMD explicitly used the terminology “fracture”. Grading was only noted in a small percentage. Variability in diagnostic interpretation by clinicians may lead to differing management plans. As identification of vertebral fracture is a trigger for treatment, developing reporting guidelines for paediatric vertebral fracture assessment will improve care. A structured template should be introduced for radiological reporting of paediatric vertebral fracture assessment.

## Introduction

Duchenne muscular dystrophy (DMD) is a rare X-linked condition presenting in early childhood with estimates of 1 in 3,500 live male births affected [1]. The use of long-term oral glucocorticoid as a disease modifier has been adopted as standard of care worldwide for over 20 years, with documented improvement in health outcomes such as prolonging ambulation, improving cardiorespiratory outcomes, and reducing the need for surgery for severe scoliosis [2, 3]. Its use, however, is associated with a range of significant side effects with fragility fractures and osteoporosis being extremely common. With long-term follow-up, approximately 75% of patients will sustain a long bone and vertebral fracture [4–6]. Therefore, early diagnosis of osteoporosis in this group of young people is imperative to allow initiation of bisphosphonate therapy in line with the current 2018 international standard of care [7].

Whilst the definition of osteoporosis in adults relies to a great extent on dual-energy absorptiometry (DXA)-based bone density thresholds, clinical diagnosis of paediatric osteoporosis takes a fracture centric approach. Both the 2013 International Society of Clinical Densitometry guidance (used in this study) [8] and the updated 2019 guidance [9] state that in growing children the identification of a single vertebral fracture is sufficient for the clinical diagnosis of osteoporosis regardless of bone density. The 2018 Care Considerations for DMD recommend routine lateral thoracolumbar spine imaging as part of bone monitoring to identify vertebral fracture, moving the focus to vertebral fracture for diagnosis of osteoporosis [7]. The 2018 International Care Considerations for DMD also recommend initiation of bisphosphonate therapy upon identification of moderate or severe vertebral fracture even if asymptomatic [7]. Mild vertebral fractures are treated if there is evidence of back pain [7]. Therefore, accurate and prompt radiological identification of vertebral fracture is crucial to improve clinical care, and to streamline management across sites.

Whilst the expertise to identify vertebral fracture on radiological imaging is important, it is known that “ambiguous” terminology used in reports (for example, wedging, reduction in vertebral height) may lead to poor communication of the accurate diagnosis. In the United Kingdom (UK), the Royal College of Radiology and the Royal Osteoporosis Society developed joint guidance on radiological reporting of vertebral fracture that includes the need for use of clear terminology (i.e. fractures) instead of ambiguous terminology (for example, reduction in vertebral height); and for grading of vertebral fracture using the Genant semi-quantitative method [10, 11]. We believe that it is especially important that a radiological diagnosis of vertebral fracture in children and adolescents is made clear by using explicit terminology, as there are also known physiological changes in growing children identified on spine radiographs that do not constitute fracture [12, 13].

This present study is an audit of (1) radiological reporting of vertebral fracture in boys with DMD in accordance with standards set out by the Royal College of Radiology and the Royal Osteoporosis Society in relation to the use of clear terminology and (2) grading of fractures (which was also part of a national audit in the UK [10, 11, 14]). We also aimed to evaluate the potential influence of radiology reports on clinical management by treating clinicians, and the impact on clinical management when clear terminology with a structured reporting template is used.

## Methods

Out of a total of 104 boys with DMD managed in two tertiary paediatric neuromuscular centres in 2019 with median age of 11.2 years (range 2.0, 17.0), 84 (81%) underwent lateral thoracolumbar spine imaging for vertebral fracture monitoring. Lateral thoracolumbar spine imaging of these 84 boys (71 lateral spine radiographs [Samsung GC80, Suwon, South Korea; and Phillips Digital Diagnost 2.1, Amsterdam, Netherlands]) and 13 lateral spine dual-energy absorptiometry images on i-DXA (GE Healthcare Lunar iDXA, Buckinghamshire, UK) were included. All lateral spine images were reported by 13 consultant paediatric radiologists with 2 to > 10 years of experience, subsequently referred to as a “radiology report”. At the time of the audit (2019), neither site had departmental guidance on reporting of vertebral fractures and therefore no formal standardisation of radiological diagnosis of vertebral fracture and grading of vertebral fracture.

The first part of this study aimed to audit the radiology reports against reporting standards laid out by the Royal College of Radiology and the Royal Osteoporosis Society [10, 11]. The standards include the use of clear recommended terminology “vertebral fracture” in relation to described abnormalities and a comment on the severity of vertebral fracture [10, 11]. The standards were also used in a recent national UK audit of radiology reporting of vertebral fractures in adults [14]. A single observer (S.C.W., a consultant paediatric endocrinologist with experience in metabolic bone with 10 years of experience) reviewed the anonymised radiology reports of the 84 lateral spine images to identify if the terminology “fracture” (i.e. either vertebral fracture or no vertebral fracture) was used and if fracture grading was provided where appropriate. For purposes of this audit, fracture grading was deemed necessary if any abnormalities in vertebral bodies and/or end-plate abnormalities were described.

The second part of the study aimed to report the clinical diagnosis made by treating clinicians based on the radiology reports of lateral spine images. Anonymised radiology reports of the 84 boys were circulated to two neuromuscular clinicians (S.J., a consultant paediatric neurologist with 4 years of experience, and J.D. a neuromuscular nurse consultant with 11 years of experience as a neuromuscular nurse specialist and a year of experience as a neuromuscular nurse consultant) and two consultant paediatric endocrinologists with experience in metabolic bone disorders (A.M. and A.K., each with 10 years consultant experience). The four clinicians did not review the lateral spine radiographs or lateral spine images on DXA. The four clinicians were asked to determine if there was vertebral fracture, no vertebral fracture, or unclear interpretation based solely on the radiology reports. The endocrinologists were also asked if bisphosphonate therapy was indicated. The two endocrinologists were aware of the 2018 international standards of care for DMD in regard to management of osteoporosis.

The third part of the study aimed to report the clinical diagnosis made by treating clinicians based on a structured report which incorporates recommendations of the Royal College of Radiology and the Royal Osteoporosis Society. The structured report specifically included information on vertebral bodies that were visualised and evaluated, explicit use of the terminology “fracture” (i.e. either vertebral fracture or no vertebral fracture), and grading of fracture. A single observer (S.C.W.) performed vertebral fracture assessment, using the Genant semi-quantitative method, in 37 out of the 84 images and re-reported them using the structured format (subsequently referred to as “structured report”). In the structured reports, vertebral fractures were graded according to the Genant semiquantitative method as follows: 20–25% height reduction (grade 1, mild fracture), >25–40% height reduction (grade 2, moderate fracture), >40% height reduction (grade 3, severe fracture). The structured reports were re-circulated to the four clinicians for their clinical diagnosis (i.e. vertebral fracture, no vertebral fracture, or unclear interpretation) and to the two endocrinologists for their opinion on management with bisphosphonate. Again, the four clinicians did not review the lateral spine radiographs or lateral DXA images.

Continuous data was expressed as median (range). Complete concordance was taken as a diagnosis of vertebral fracture or no vertebral fracture between the four clinicians. This study was conducted as a clinical audit against established standards and all data were completely anonymised for purposes of the audit.

## Results

### Part 1: auditing the use of clear terminology and grading of fractures in unstructured clinical radiology reports

**Fig. 1:**
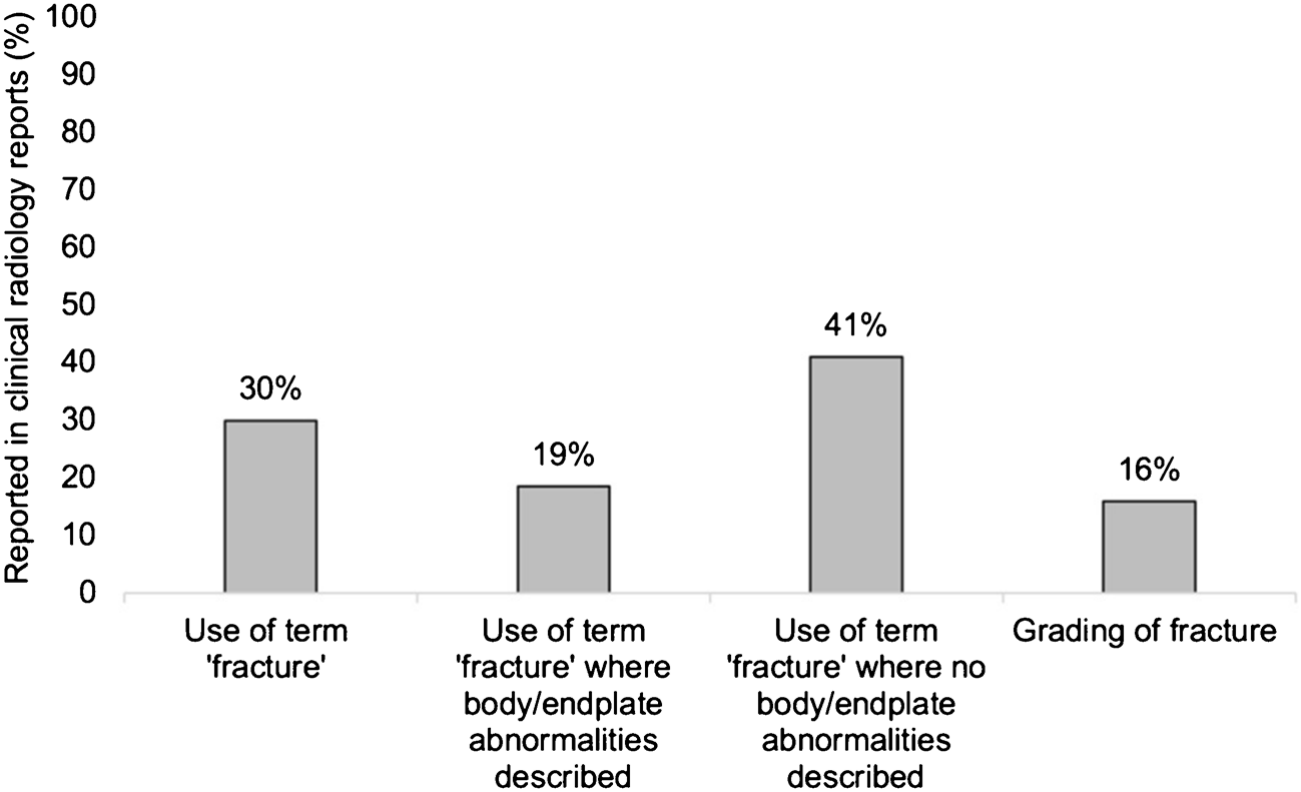
Terminology used in unstructured clinical radiology reports

Abnormalities in vertebral bodies and/or end-plates were reported in 43/84 (51%) radiology reports. The term “fracture” was explicitly used in 25/84 (30%) reports and only in 8/43 (19%) with description of vertebral bodies and/or end-plates abnormalities. Terminologies like “some mild reduction in height” and “some end-plate changes” were used in the remaining reports. Of those where abnormalities were reported, 7/43 (16%) included grading of vertebral abnormalities (Fig. 1).

### Part 2: evaluating clinicians’ diagnostic interpretation of unstructured clinical radiology reports

Based on radiology reports, the first clinician identified 15/84 (18%) individuals with fracture, 38/84 (45%) with no fracture, and 31 (37%) in which diagnosis was deemed unclear. The second clinician identified 9/84 (11%) individuals with fracture, 46/84 (55%) with no fracture, and 29/84 (35%) unclear diagnoses. The third clinician identified 21/84 (25%) individuals with fracture, 43/84 (51%) with no fracture, and 20/84 (24%) unclear diagnoses. The fourth clinician identified 26 (31%) individuals with fracture, 35/84 (42%) with no fracture, and 23/84 (27%) unclear diagnoses. Complete concordance in diagnostic interpretation by the four clinicians was only noted in 36/84(43%), and only in 7/43 (16%) where abnormalities in vertebral bodies and/or end-plate changes were described. Interpretation of radiology reports by the first endocrinologist would have resulted in initiation of bisphosphonate treatment in 10/84 (12%), and by the second endocrinologist in 9/84 (11%). In 37/84 (44%) and 15/84 (18%), the endocrinologists were unable to make a management recommendation. Concordance in treatment plans with bisphosphonates (i.e. treatment or no treatment) was observed in 45/84 (54%). In all the 45 where concordance in treatment plans with bisphosphonates between the two endocrinologists was reported, concordance in diagnostic interpretation of whether there was vertebral fracture or no vertebral fracture was also reported.

### Part 3: evaluating clinicians’ diagnostic interpretation of structured re-reported reports

**Fig. 2:**
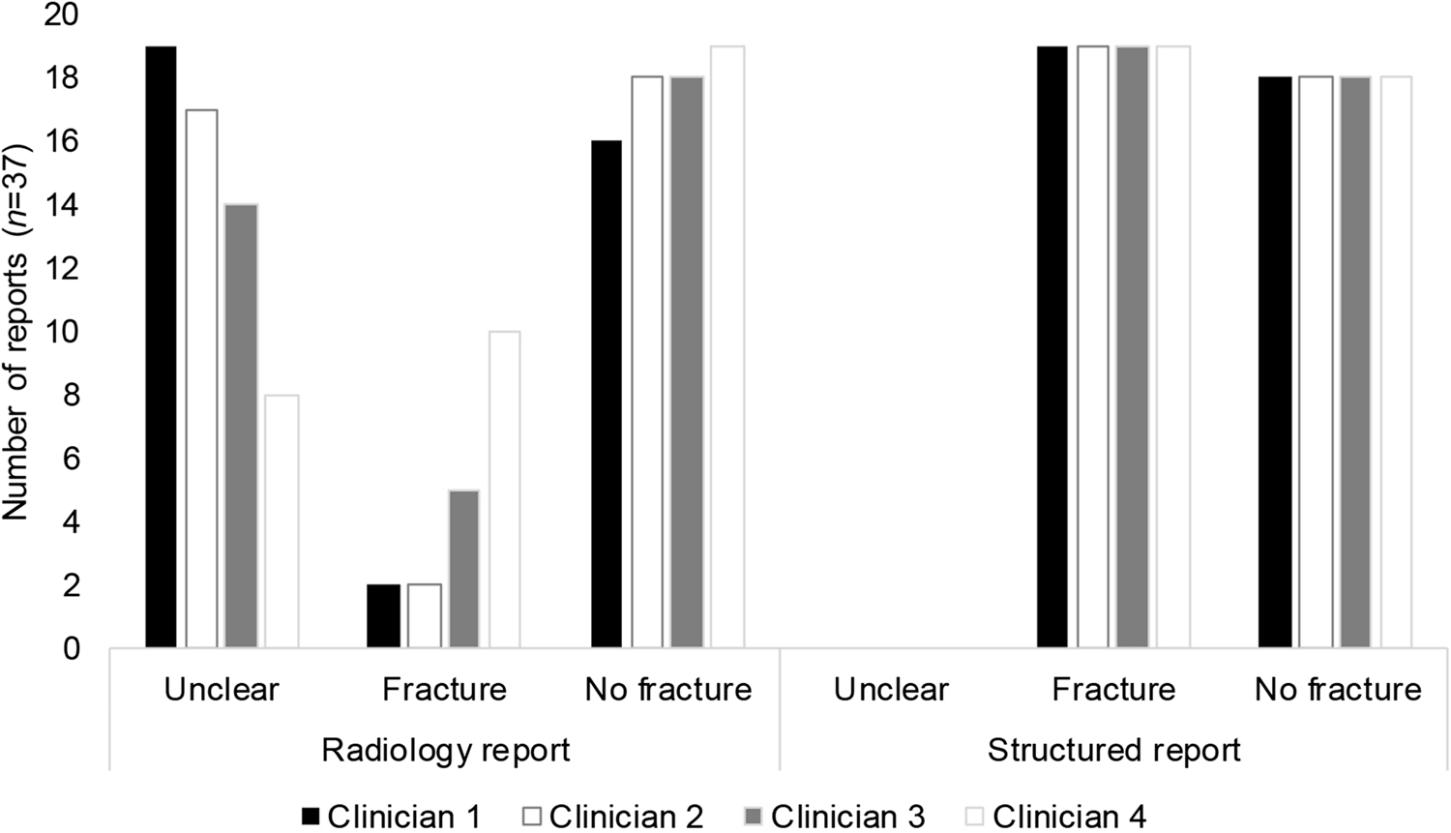
Clinician interpretation of radiology (unstructured) and structured reports. Direct comparison of clinician interpretation of the 37 reports that were re-reported in structured format. Clinicians determined whether there was fracture, no fracture, or unclear interpretation based on each report

**Fig. 3:**
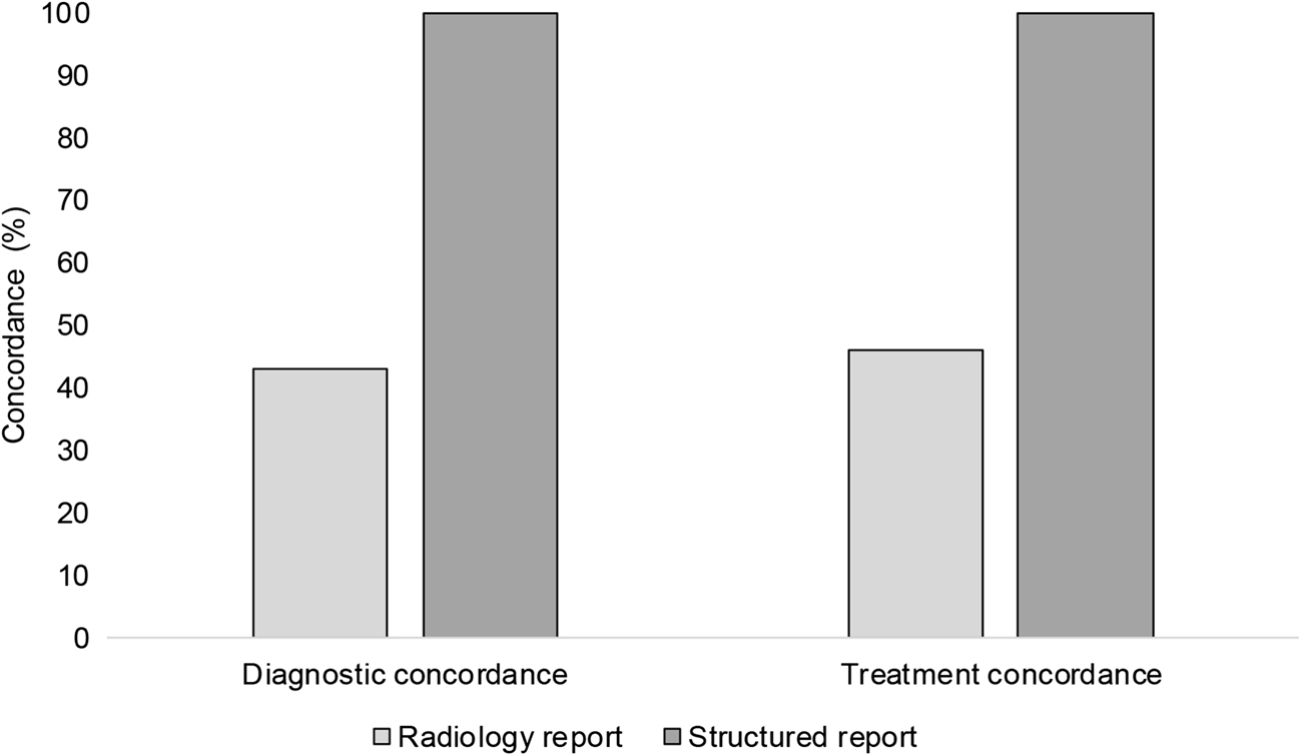
Diagnostic and treatment concordance between clinicians. Direct comparison of diagnostic and treatment concordance by the four clinicians based on radiology and structured reports. Diagnostic concordance defined as all 4 clinicians arriving at the same diagnosis of fracture, or no fracture. Treatment concordance defined as both endocrinologists arriving at the same treatment decision of bisphosphonate or no bisphosphonate

Based on the structured reports (*n*=37), all four clinicians were completely concordant in the diagnosis of fracture (or no fracture). Interpretation of the 37 structured reports demonstrated 19 (51%) with fracture, 18 (49%) with no fracture, and none with unclear interpretation by all four clinicians (Fig. 2). Complete concordance in treatment plans with bisphophonates was noted when the two endocrinologists reviewed the structured reports. Interpretation of the structured reports by both endocrinologists would have resulted in initiation of bisphosphonate treatment in 13/37 (35%). For direct comparison, interpretation of the radiology reports of the 37 images included in this part of the study demonstrated diagnostic concordance in 16/37 (43%), and treatment concordance in 17/37 (46%) (Fig. 3).

## Discussion

This dual centre audit of lateral thoracolumbar spine images (radiographs and lateral dual-energy absorptiometry) for vertebral fracture in boys with DMD against standards set out by the Royal College of Radiology and the Royal Osteoporosis Society in the UK highlights several issues which are important for clinical care [10, 11]. Only a third of radiology reports used the word “fracture” explicitly; and this was only in under 20% of reports where vertebral body and/or end-plate abnormalities were described. In our present audit, grading of vertebral fracture was only included in less than 20% of radiology reports where abnormalities were described. As the current 2018 International Standards of Care recommend treatment with bisphosphonate in DMD based on grading of vertebral fracture (i.e. only moderate or severe vertebral fracture), “ambiguous” terminology and lack of information on grade may lead to variability in clinical management [7]. Moreover, as ongoing clinical decisions about management with bisphosphonates take into consideration the vertebral fracture phenotype (incident new vertebral fracture with treatment or worsening of grade of vertebral fracture), the need for radiological information to be relayed to treating clinicians is important beyond the diagnosis of osteoporosis. Our audit suggests that a structured reporting template for vertebral fracture assessment should be used in paediatric clinical practice.

The role of clear rather than ambiguous terminology (for example, reduction in or loss of vertebral height, wedging or deformity) is paramount in ensuring that accurate radiological diagnosis is communicated effectively to managing clinicians. Our audit identified that the term “fracture” was explicitly used in only 19% (target of 100% laid out by Royal College of Radiology and Royal Osteoporosis Society audit standards [14]) of images with abnormalities described. Only 16% (target of 100% laid out by Royal College of Radiology and Royal Osteoporosis Society audit standards [14]) of those with abnormalities described included grading of fracture with potential impact on clinical diagnosis and management in boys with DMD given the 2018 international standards of care [7]. By comparison, in a recent national audit of vertebral fracture reporting using CT images in adults, the term “fracture” was used explicitly in 60% of images and 26% included grading of fracture [14]. As there are known normal variants of the vertebrae during growth (like physiological wedging, anterior beaking, Schmorl’s nodes) and other non-vertebral fracture conditions, summarised in the pictorial review by Jaremko et al. [12], clearly communicating if the abnormalities identified are fractures or such normal variants is crucial. Our audit also showed that clinical diagnosis based on current radiology reports, with ambiguous terminology, may differ between clinicians and with differing treatment plans.

Longitudinal natural history studies of glucocorticoid-treated children with chronic disorders like acute lymphoblastic leukaemia and chronic rheumatic disorders show that vertebral fractures are very common and indeed a powerful predictor of incident vertebral fracture and long bone fractures [15, 16]. Clinical fragility fractures have been variably reported as occurring in between 50% and 75% of boys with DMD [5, 6]. Vertebral fracture is identified in about 30–40% of boys on daily glucocorticoid when routine lateral spine imaging is performed [17, 18]. The 2018 International Standards of Care represents a significant step change in management of osteoporosis in these young people in that it recommends initiation of intravenous bisphosphonate therapy in boys with evidence of vertebral fracture of at least moderate grade (Genant 2) even if asymptomatic [7]. The recommendation of the use of intravenous bisphosphonate is supported by the results of a recent randomized controlled trial of intravenous bisphosphonates in boys with DMD where treated boys showed improvement in bone density with fewer boys with severe vertebral fracture after two years of therapy [19]. Therefore, we believe that accurate identification and clear reporting of vertebral fracture, including grade, can improve clinical care with timely initiation of bisphosphonates to prevent future fractures, back pain, and spine deformities. Lack of clear terminology in reports can result in diagnostic uncertainty with impact on management decisions, highlighted by our results whereby endocrinologists were unable to make management recommendations in 18–44% of radiology reports.

Our audit showed that structured reporting with clear use of fracture terminology and grading resulted in consistent diagnostic and management decisions between clinicians. When treating clinicians reviewed results of the 37 images that were reported both by radiology teams (radiology report) and in structured format (structured report), there was complete diagnostic concordance in almost 40%, and complete treatment concordance in just under 50% for the radiology reports as compared to 100% diagnostic and 100% treatment concordance with the structured reports. In the absence of this structure and clarity, 11/37 (30%) would not have been treated with bisphosphonate (either unable to make management decision or clinical recommendation of no bisphosphonate even with identification of vertebral fracture), whereas on review of structured reports would have been treated with bisphosphonate. This represents a distinct discordance in management and would have the potential to affect long-term fracture outcomes in these boys. We believe that a structured reporting template for paediatric vertebral fracture assessment would improve communication and clinical care. Neither site included in our audit have a structured template for reporting vertebral fracture. However, it is worth noting that only 6% of sites in the recent national adult vertebral fracture reporting audit had a departmental structured reporting template [14]. Formal guidance at a national or international level engaging with all relevant stakeholders including paediatric reporting and managing clinicians will be very helpful. We provide examples of two structured templates, generated following this audit, which may be incorporated into routine clinical reporting (Tables 1 and 2). The template for reporting used in our audit was similar but not identical to the template in Table 2. Our study template did not include patient identifiable information, adequacy of patient positioning or recommendations.

**Table 1:**
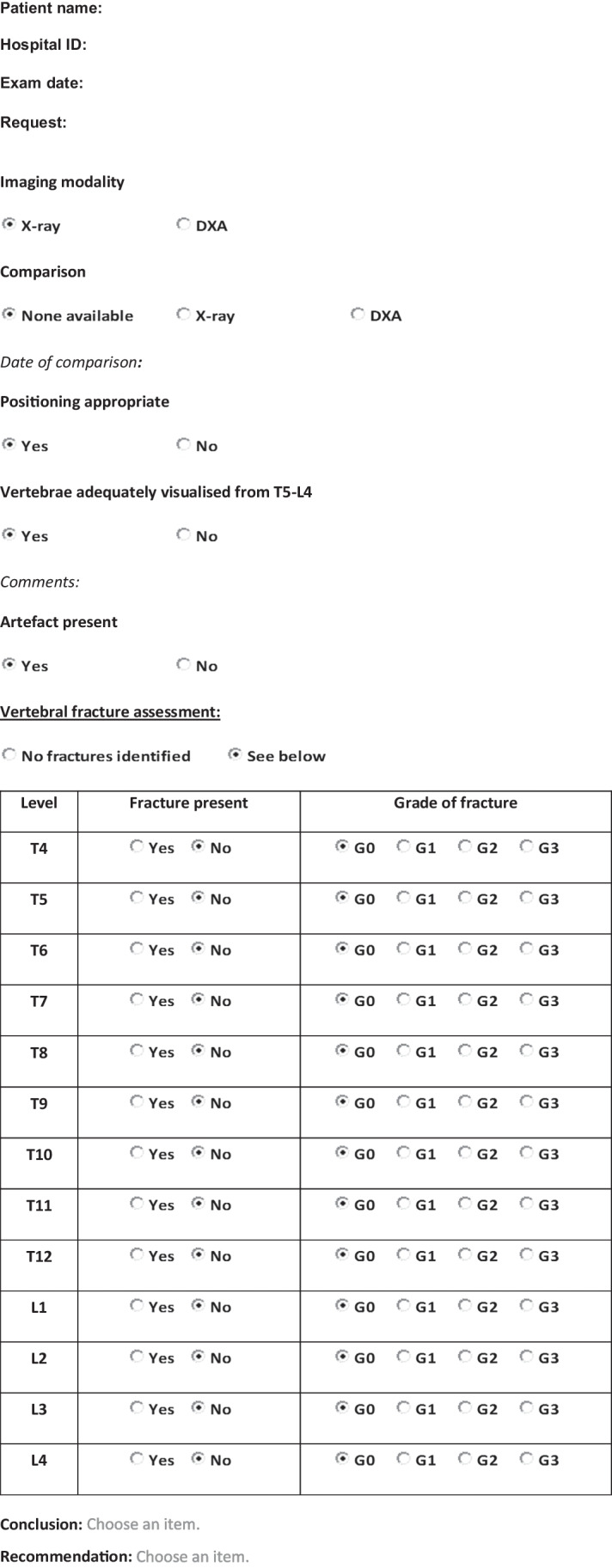
Example of reporting template for vertebral fracture assessment

**Table 2:**
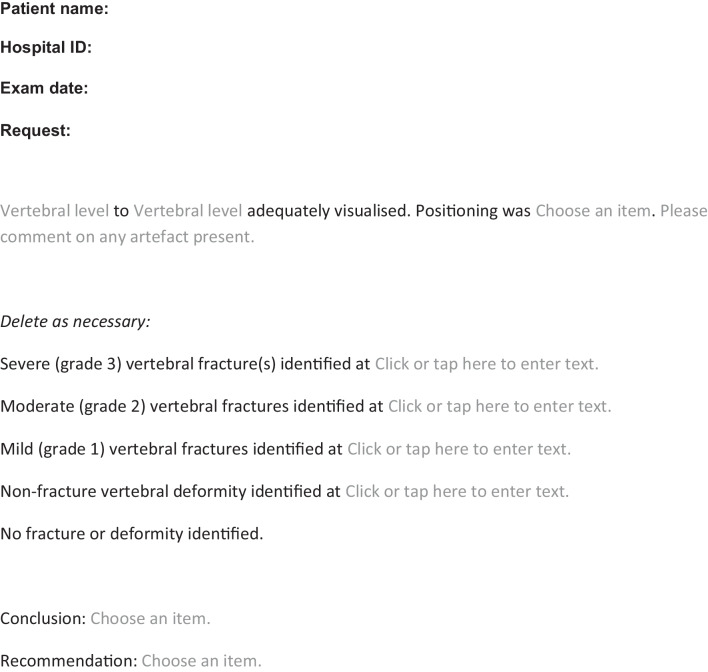
Example of reporting template for vertebral fracture assessment

There may be several possible reasons for the lack of compliance with the Royal College of Radiology and Royal Osteoporosis Society audit standards in our study. It is likely that paediatric radiologists are not aware of these guidance and audit standards. Some reporting clinicians may also be unaware of the clinical importance of identification of vertebral fracture and implications for initiation of bisphosphonate therapy, especially in DMD. Expertise in the recognition of vertebral fracture is no doubt an area that requires further upskilling within paediatric services as a whole, given that vertebral fracture is relatively uncommon in children. A study of vertebral fracture reporting in adults showed that vertebral fracture is frequently under-reported especially by non-musculoskeletal radiologists [20]. Not all clinical reports included in our audit were reported by paediatric radiologists with specific musculoskeletal interest or expertise. Developing learning modules on vertebral fracture recognition and reporting could be an important step to improve skills in this area. The International Society of Clinical Densitometry and Royal Osteoporosis Society have online educational modules for vertebral fracture recognition, although both are not paediatric focussed. The development of a paediatric vertebral fracture assessment learning module should now be considered.

The present study is an audit aiming to evaluate the impact of clear terminology of vertebral fracture reporting and its potential impact on clinical management. Another very important issue beyond the scope of our study is the definition of paediatric vertebral fracture. In our study, we used the semi-quantitative method of Genant as recommended by the International Society for Clinical Densitometry for diagnosis of paediatric vertebral fracture [9]. Using the semi-quantitative Genant method for diagnosis of paediatric vertebral fracture, the Canadian Steroid-associated Osteoporosis in the Paediatric Population (STOPP) Consortium provided validation that the threshold of a >20% loss of height ratio in a clinical research setting could be an appropriate criterion for the diagnosis of vertebral fracture as vertebral fractures diagnosed based on those criterion were linked with biologically important clinical end-points such as back pain, lumbar spine bone mineral density *Z*-scores, and second metacarpal cortical area *Z*-scores [16, 21–23]. In addition, the investigators identified that vertebral fractures diagnosed using the Genant semi-quantitative method predicted subsequent long bone fractures in children with leukaemia [21] and subsequent vertebral fractures in children with leukaemia and DMD [21, 24]. However, it has to be highlighted that the semi-quantitative method of Genant was developed based on spine radiographs in postmenopausal women [25], and there are therefore some concerns about its use in the paediatric population. A significant limitation of the semi-quantitative method is that end-plate abnormality is not accounted for; and dependent on the experience of the observer, normal variants maybe misdiagnosed as mild vertebral fracture. The challenges and validity of the diagnosis of mild vertebral fracture in clinical practice using the semi-quantitative method should also be acknowledged. Further research into other classification systems for the diagnosis of paediatric vertebral fracture like algorithm-based qualitative methods [26, 27] that are easy to use in clinical practice and with high inter-observer agreement is needed. Regardless of the classification system used for diagnosis of paediatric vertebral fracture, we believe that clear reporting would still be important.

There are of course limitations to our audit in that we included a relatively small number of patients/images (84) and only from two sites. The Royal Osteoporosis Society recommends an adequate audit sample size of 150–200; however, this pertains to adults with osteoporosis which is a more frequently encountered condition than children with osteoporosis. The managing clinicians in our study were asked for their clinical diagnosis based on their interpretation of the site radiology reports of lateral spine images only, without any clinical information and indeed did not review the patients—therefore did not reflect real life practice. Images were re-reviewed and reported in a structured format by a clinician with expertise in paediatric osteoporosis and vertebral fracture assessment. We have, therefore, not compared non-standardised (radiology report) and standardised reports (structured report) by radiologists.

## Conclusion

In summary, in this first audit of vertebral fracture reporting in children and adolescents, under 20% of radiology reports of lateral spine imaging in DMD with vertebral abnormalities explicitly used the terminology “fracture”. Grading was also only noted in a small percentage of radiology reports. We show the potential for differing clinical diagnosis and treatment plans by treating clinicians based on the radiology reports. As the identification (and grading) of vertebral fracture is a trigger for treatment, developing reporting guidelines for paediatric vertebral fracture assessment including consensus of clear use of terminology and the introduction of structured reporting templates could be steps to streamline clinical care across sites. Further research into an alternative classification system for paediatric vertebral fracture that is easy to use in clinical practice, with high inter-observer agreement and which accounts for physiological variation during growth, is greatly needed.

## Data Availability

The datasets generated during and/or analysed during the current study are available from the corresponding author on reasonable request.
